# Limits on the reproducibility of marker associations with southern leaf blight resistance in the maize nested association mapping population

**DOI:** 10.1186/1471-2164-15-1068

**Published:** 2014-12-05

**Authors:** Yang Bian, Qin Yang, Peter J Balint-Kurti, Randall J Wisser, James B Holland

**Affiliations:** Department of Crop Science, North Carolina State University, Raleigh, NC 27695 USA; Department of Plant Pathology, North Carolina State University, Raleigh, NC 27695 USA; U.S. Department of Agriculture-Agricultural Research Service, Plant Science Research Unit, Raleigh, NC 27695 USA; R.J. Wisser, Department of Plant and Soil Sciences, University of Delaware, Newark, DE 19716 USA

**Keywords:** Quantitative trait loci, Nested association mapping, Disease resistance, Genome wide association study, *Zea mays*

## Abstract

**Background:**

A previous study reported a comprehensive quantitative trait locus (QTL) and genome wide association study (GWAS) of southern leaf blight (SLB) resistance in the maize Nested Association Mapping (NAM) panel. Since that time, the genomic resources available for such analyses have improved substantially. An updated NAM genetic linkage map has a nearly six-fold greater marker density than the previous map and the combined SNPs and read-depth variants (RDVs) from maize HapMaps 1 and 2 provided 28.5 M genomic variants for association analysis, 17 fold more than HapMap 1. In addition, phenotypic values of the NAM RILs were re-estimated to account for environment-specific flowering time covariates and a small proportion of lines were dropped due to genotypic data quality problems. Comparisons of original and updated QTL and GWAS results confound the effects of linkage map density, GWAS marker density, population sample size, and phenotype estimates. Therefore, we evaluated the effects of changing each of these parameters individually and in combination to determine their relative impact on marker-trait associations in original and updated analyses.

**Results:**

Of the four parameters varied, map density caused the largest changes in QTL and GWAS results. The updated QTL model had better cross-validation prediction accuracy than the previous model. Whereas joint linkage QTL positions were relatively stable to input changes, the residual values derived from those QTL models (used as inputs to GWAS) were more sensitive, resulting in substantial differences between GWAS results. The updated NAM GWAS identified several candidate genes consistent with previous QTL fine-mapping results.

**Conclusions:**

The highly polygenic nature of resistance to SLB complicates the identification of causal genes. Joint linkage QTL are relatively stable to perturbations of data inputs, but their resolution is generally on the order of tens or more Mbp. GWAS associations have higher resolution, but lower power due to stringent thresholds designed to minimize false positive associations, resulting in variability of detection across studies. The updated higher density linkage map improves QTL estimation and, along with a much denser SNP HapMap, greatly increases the likelihood of detecting SNPs in linkage with causal variants. We recommend use of the updated genetic resources and results but emphasize the limited repeatability of small-effect associations.

**Electronic supplementary material:**

The online version of this article (doi:10.1186/1471-2164-15-1068) contains supplementary material, which is available to authorized users.

## Background

Methods for elucidating the genetic architecture underlying quantitative variation in plants have evolved substantially over the last 25 years, following the first report of genome-wide quantitative trait locus (QTL) mapping [1]. The maize nested association mapping (NAM) population is composed of 5,000 recombinant inbred lines (RILs) derived from crosses between inbred line B73 and 25 other inbred lines of maize [2]. These parents were selected to capture a maximum amount of molecular genetic diversity present across the major subpopulations of public maize breeding germplasm [3, 4]. The maize NAM population has been used to study genetic architectures for a number of quantitative traits of maize [5–11], including Southern leaf blight (SLB) resistance [8].

Southern leaf blight is a foliar disease of maize caused by the fungus *Cochliobolus heterostrophus*. The disease was responsible for a major epidemic in the U.S. in the 1970’s [12] and continues to limit or threaten maize production worldwide. Natural variation in resistance to SLB is polygenic and may involve a diverse array of functional genes and pathways [8, 13]. Using joint linkage mapping (JLM) and genome-wide association study (GWAS) the genetic architecture of resistance to SLB in the NAM population was associated with more than 30 loci with small additive effects [8]. With the recent release of maize HapMap2 [14] and a denser linkage map based on genotyping-by-sequencing (GBS [15, 16]) with markers positioned every 0.2 cM, QTL identified by JLM can be more precisely localized on the genetic and physical sequence maps. The denser linkage map is also expected to permit more accurate projection of the more than 28 M SNPs among parental lines in maize HapMaps 1 and 2 onto NAM RILs, which should provide mapping precision to the limits dictated by linkage and disequilibrium in this population.

Two-stage regression analysis has been widely used to test SNPs for associations with quantitative diseases in human [17–19], and this approach has been adopted for GWAS in plants. In the first stage, observed phenotypes are regressed on covariates such as demographic, clinical, and/or environmental factors. In the second stage, the residual values from the first stage model (‘residual outcomes’ or adjusted phenotypic values) are regressed on genetic markers with simple- or multiple-linear regression. Despite its convenience in computation, the two-stage method can result in a downwardly biased estimate of genotypic effects and loss of power in detection as a result of dependency between covariates and the tested SNP genotypes [20, 21]. Two-stage approaches are also used to combine the complementary advantages of JLM and GWAS in NAM [7, 8, 10]. In the first stage, JLM is performed using a consensus linkage map to identify QTL across the genome. In the second stage, GWAS is performed chromosome-by-chromosome, using separate input values for each chromosome that are obtained as residuals from the first stage QTL model, built by excluding QTL on the chromosome to be tested for GWAS. The purpose of this is to adjust phenotype values used for association analysis for the effects of QTL on other chromosomes. This approach is expected to be largely free of the problem of dependency between covariates (QTL) fit in the first stage and SNPs tested in the second stage, since only QTL on different chromosomes than the test SNPs are fit as covariates.

We are working toward identifying the causal variants underlying quantitative resistance to SLB, relying, in part, on the information provided by NAM. The objective of this study was to re-analyze resistance to SLB in the maize NAM panel using the updated genetic and haplotype maps, to compare the results with those of the previous analysis, and to determine which results are more reliable. The previous JLM analysis was based on SLB phenotypes measured on 4694 RILs and a linkage map of 1106 SNPs [2], and the previous GWAS analysis was based on 1.6 M SNPs of HapMap 1 [22]. Since that analysis, the mixed model used to produce the phenotypic inputs to the analysis was updated to better adjust for the effect of flowering time on SLB resistance phenotypes. The updated 7386-marker map has a uniform density of one marker every 0.2 cM, but the number (4413) of RILs phenotyped and genotyped with this map is smaller than previously (4694 RILs). Therefore, a second objective of this study was to measure the influence of each of the changes in the data used for analysis (genetic maps, RIL sample sizes, and phenotype values) on the current two-stage JLM-association analysis in the NAM panel, using SLB as an example. Finally, cross-validation was used to compare the prediction power of the original JLM model of Kump et al. [8] and the updated JLM model in the NAM panel.

## Results

### Modeling the effect of flowering time on SLB resistance

The statistical association between flowering time and disease resistance was complex. Among the 135 of 156 possible combinations of rating × environment × NAM populations for which there were sufficient data for analysis, there was no significant relationship between flowering time and SLB resistance for 56 combinations, linear relationships for 75 combinations, and quadratic relationships for 4 combinations (Additional file 1: Table S1). Only four populations exhibited a consistent relationship across ratings and environments (no effects for populations 11 and 22; linear effects for populations 8 and 26). Thus, the majority of disease ratings in every population exhibited significant but variable relationships with flowering time. The flowering time covariate effects were generally small (*r*^2^ ranged from 1% to 22%), however, and the updated combined mixed model incorporating variation in the flowering time covariate effect only slightly altered the BLUPs: the original and updated BLUPs were highly correlated between entries in each population (*r*_min_ =0.980; *r*_max_ =0.997), and there were only subtle differences in the rankings of population mean effects (*r*_s_ =0.983).

### Precision of QTL localization and improved QTL prediction power

**Figure 1:**
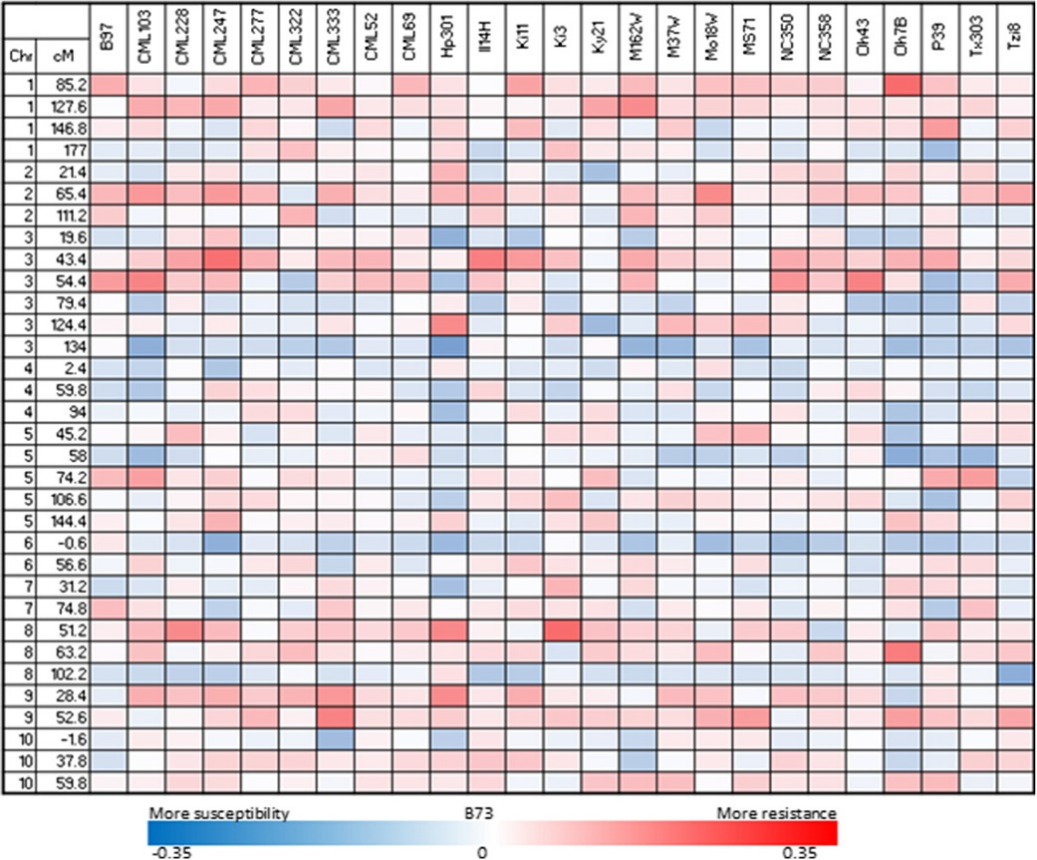
**Heat map of additive effect estimates of 25 founder parent alleles for QTLs of SLB resistance relative to B73.** QTL are identified by their genetic locations in the consensus genetic map (7386-marker map); effect estimates for each parental allele are indicated by color blocks. Negative cM values for markers indicate that they are distal to the first marker from the original NAM linkage map on that chromosome.

JLM analysis with the 7386-marker map, updated BLUPs of 4413 RILs, and iterative optimization (model 7 in Table 1), identified 33 QTL (referred to as ‘model 7 QTL’) associated with variation in resistance to SLB in the NAM panel, with support intervals averaging 4.6 cM and ranging from 1.8 cM to 14.0 cM (Table 2). Combined, the 33 QTL from model 7 were associated with 84% of the phenotypic and 98% of the genotypic variation for resistance to SLB. All model 7 QTL had small effects; absolute values of significant (*p* <0.05) allele effects averaged 0.14 (range: 0.07 to 0.35) on the 1–9 scale used for quantifying resistance [8] (Figure 1; Additional file 1: Tables S2 and S3). The two model 7 QTL with the largest resistance effects across RIL families mapped to 43.4 cM and 54.4 cM on chromosome 3.

**Table 1:** **Inputs for joint linkage mapping QTL analysis and GWAS models**

	Joint linkage mapping QTL models		
QTL Model	Phenotype	No. markers in linkage map	No. NAM RILs
1^a^	original^b^	1106	4694
2	original	1106	4354
3	updated	1106	4694
4	updated	1106	4354
5	original	7386	4354
6	updated	7386	4354
7	updated	7386	4413
**GWAS models**			
**GWAS Model**	**QTL model used to adjust phenotypes**	**Variants tested for association**	***p*** **value threshold**
A^a^	1	1.6 M HM1 SNPs	1E-4
B	1	1.6 M HM1 SNPs	1E-7
C	1	28.2 M HM1/2 SNPs + 0.2 M RDVs	1E-7
D	7	1.6 M HM1 SNPs	1E-7
E	7	28.2 M HM1/2 SNPs + 0.2 M RDVs	1E-7

^a^QTL model 1 and GWAS model A results were previously published [8].

^b^Original phenotype inputs were RIL BLUPs across 3 environments, based on a model with a common flowering time covariate; updated phenotype inputs were RIL BLUPs across 3 environments based on a model with environment-specific flowering time covariates.

**Table 2:** **Physical and genetic positions for QTL peaks and support intervals (SI) mapped using updated phenotypes and linkage map (model 7) and comparison to QTL previously reported by Kump et al.** [8] **(model 1)**

Chr	Peak position (AGP v2 bp)	Peak position (cM)	SI Map Position (cM)	Step included	SI overlapped	Distance between QTL peaks (cM)
1	90,443,174	85.2	84.2 - 86.2	3	y	0.8
1	218,082,692	127.6	125.6 - 129.6	8	y	1.9
1	251,723,948	146.8	144.8 - 147.8	18	y	1.0
1	283,549,061	177	175 - 179	22	n	29.2
2	7,180,393	21.4	19.4 - 23.4	21	n	20.2
2	36,838,070	65.4	64.4 - 66.4	5	y	2.3
2	206,275,294	111.2	110.2 - 113.2	23	n	30.8
3	5,348,237	19.6	18.6 - 22.6	13	y	5.9
3	16,246,035	43.4	41.4 - 44.4	1	n	6.6
3	31,533,927	54.4	53.4 - 55.4	10	c	4.4
3	170,374,260	79.4	78.4 - 84.4	14	n	12.9
3	214,568,867	124.4	123.4 - 126.4	26	c	6.8
3	219,498,075	134	132 - 137	6	y	2.8
4	1,892,489	2.4	-3.6 - 7.4	29	y	4.6
4	141,422,921	59.8	54.8 - 60.8	20	c	7.9
4	181,935,681	94	91 - 102	17	n	24.2
5	15,138,119	45.2	44.2 - 49.2	33	y	8.2
5	36,905,989	58	55 - 59	11	y	4.6
5	158,046,075	74.2	72.2 - 77.2	19	y	3.0
5	200,161,433	106.6	104.6 - 109.6	27	n	18.1
5	214,137,041	144.4	140.4 - 154.4	31	n	19.7
6	6,929,855	-0.6	-1.4 - 0.4	9	y	0.4
6	144,806,691	56.6	53.6 - 58.6	28	n	56.8
7	8,270,900	31.2	28.2 - 34.2	30	y	3.0
7	142,429,440	74.8	73.8 - 76.8	25	c	5.2
8	36,978,572	51.2	50.2 - 53.2	4	y	1.4
8	118,435,453	63.2	61.2 - 67.2	24	n	10.6
8	166,705,312	102.2	101.2 - 104.2	15	y	1.3
9	16,361,287	28.4	27.4 - 29.4	7	y	0.1
9	109,899,486	52.6	51.6 - 54.6	2	n	5.6
10	2,040,278	-1.6	-2.6 - 3.4	32	n	38.3
10	75,958,884	37.8	34.8 - 38.8	12	y	1.1
10	135,354,773	59.8	58.8 - 60.8	16	n	23.1

‘Step included’ denotes the regression model building step in which each QTL was selected for inclusion in model 7. ‘SI overlapped’ indicates if models 1 and 7 QTL SI overlapped: y for overlapped, n for not overlapped and c for very close (within 1.5 cM) but not overlapped. ‘Distance between QTL’ indicates the cM distance between the peaks of nearest QTL from models 1 and 7.

The JLM results are generally similar to those reported by Kump et al. [8], who identified 32 QTL (here, model 1), each of which had relatively small allelic effects of similar magnitude to model 7 QTL. To directly compare the positions of QTL from models 1 and 7, we interpolated model 1 QTL peak positions onto the 7386-marker map according to the AGP v2 physical positions of the SNPs identified as model 1 QTL peaks (Table 2). The median distance between the closest matching QTL peaks of model 1 and 7 was 5.6 cM (Table 2). Smaller-effect QTL tended to have larger discrepancies in position between the models.

Prediction accuracy of JLM QTL models developed from original and updated inputs were compared by cross-validation. A small number of RILs used in model 7 were not used in model 1 because of missing data in the original linkage map, so we identified the set of 4354 RILs in common between the original and updated data sets. QTL positions from models 1 and 7 were fit to random subsets of these RILs to re-estimate the allele effects and predict phenotypes in the validation sets. On average, across 100 randomly sampled training and validation sets, model 7 had a significantly (*p* <0.0001) greater prediction correlation coefficient (*r* =0.86 ± 0.01) than model 1 (*r* =0.83 ± 0.01; Additional file 2: Figure S1).

### Sensitivity analysis

**Figure 2:**
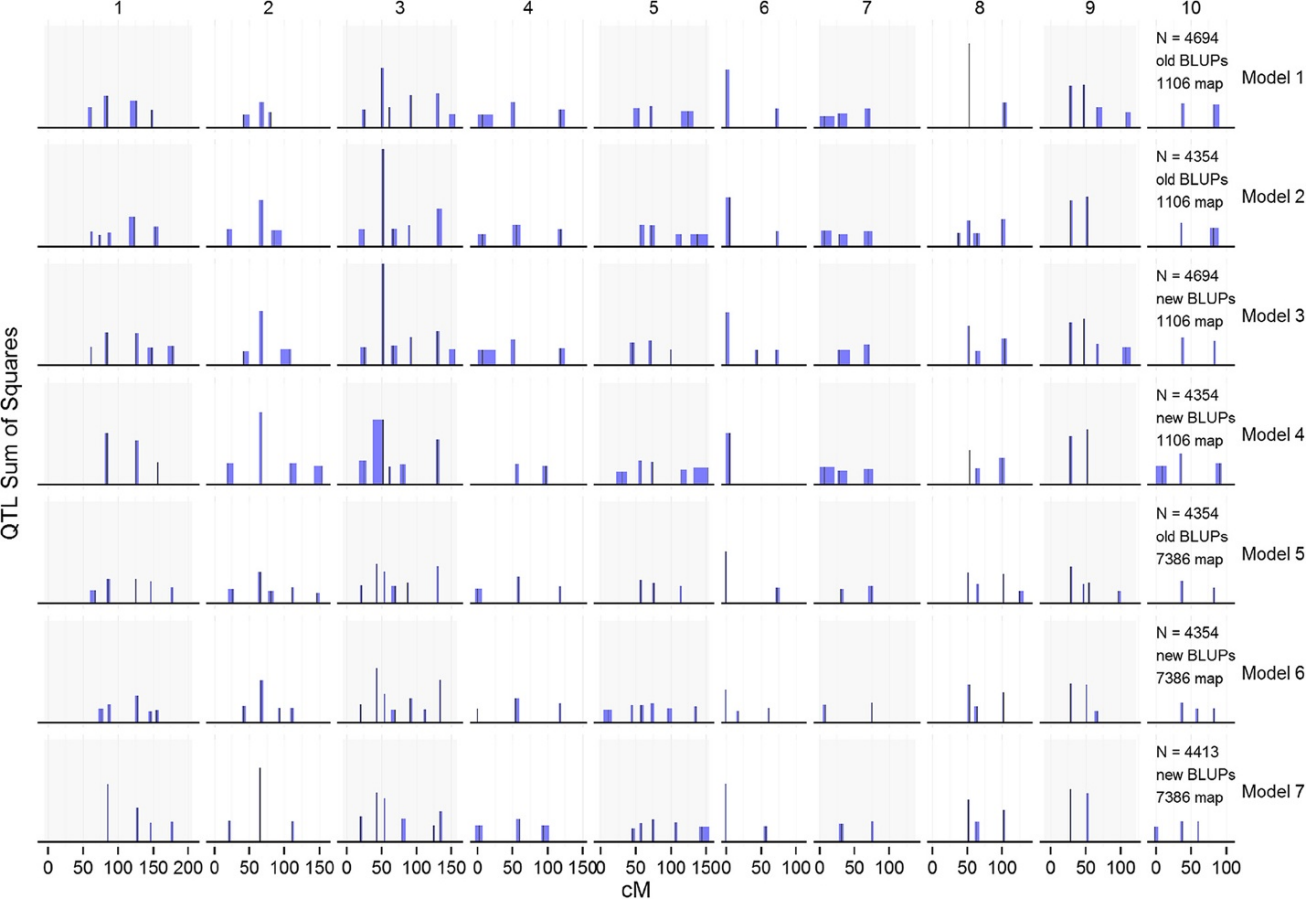
**QTL positions on the ten maize chromosomes from seven joint linkage mapping models.** Positions in cM are based on 7386-marker map. QTL bar heights are proportional to their partial *R* ^*2*^, blue-colored margins denote the QTL support intervals, and black spikes denote the QTL peak positions. For the definitions of model inputs, see Table 1.

Seven different QTL models (including models 1 and 7 previously described) were generated using different combinations of model inputs. The inputs that varied included the genetic map (1106-marker map vs. 7386-marker map), phenotypes (“original BLUPs” vs. “updated BLUPs”), and RIL sample sizes (4354 vs. 4431 vs. 4694 RILs; Table 1). QTL peak locations were generally concordant among the seven models tested (Figure 2). Predicted phenotypic values of RILs based on JLM QTL models were also similar among the models, with all correlation coefficients between model predictions greater than 0.94.

In contrast to the general stability of QTL localization and predicted phenotypic values observed among models varying for different inputs, we observed substantially lower correlations between chromosome-specific residual outcomes from these JLM QTL models (Table 3). Genetic map density (1106-marker map vs. 7386-marker map) had the greatest impact on the correlation between residual outcomes, followed by sample size (4694 vs. 4354 RILs), and then the different methods for handling the flowering time covariate in generating the RIL phenotypes (original vs. updated BLUPs). Using identical algorithms and phenotype inputs but different marker densities (1106 vs. 7386) produced a correlation of 0.79 (models 2 and 5, models 4 and 6; Table 3). Dropping 340 RILs (4694 vs. 4354 RILs) produced a correlation of 0.85 (models 1 and 2, models 3 and 4; Table 3). The decrease in correlation due to sample size was attributed to total sample size *per se* and not likely to representation of particular families, since the proportional representation of each family in the total NAM family did not change due to dropping lines (Additional file 1: Table S4). Although original and updated BLUPs were highly correlated as input values (*r* =0.99), their small differences resulted in QTL model residuals with much greater differences, reflected in correlations of 0.80 to 0.91 between residual outcomes differing only for original vs. updated BLUPs (Model comparisons 1 vs 3, 2 vs. 4, and 5 vs. 6; Table 3). When multiple inputs were changed simultaneously, the correlation between residuals diminished more (*r* ranging from 0.77 to 0.85, Table 3).

**Table 3:** **Input changes in joint linkage mapping QTL modeling affect the values of the chromosome-specific residuals more profoundly than the corresponding predicted phenotypic values**

Model	1	2	3	4	5	6
1	4694 RILs, original BLUPs, 1106 map	0.88	0.91	0.81	0.81	0.77
2	SZ	4354 RILs, original BLUPs, 1106 map	0.85	0.87	0.82	0.79
3	PH	PH, SZ	4694 RILs, updated BLUPs, 1106 map	0.82	0.79	0.79
4	PH, SZ	PH	SZ	4354 RILs, updated BLUPs, 1106 map	0.80	0.77
5	GN, SZ	GN	GN, PH, SZ	GN, PH	4354 RILs, original BLUPs, 7386 map	0.80
6	GN, PH, SZ	GN, PH	GN, SZ	GN	PH	4354 RILs, original BLUPs, 7386 map

Upper diagonal shows average correlation of residual outcome for each chromosome for 15 pairwise model comparisons. Lower diagonal shows the input(s) that differed in each pair of model comparison. “GN”, “PH”, and “SZ” denote the different genotype inputs: GN, linkage map (1106- vs. 7386-marker map); PH, phenotype inputs (original vs. updated BLUPs); and SZ, sample size (4354 vs. 4694 RILs), respectively. Diagonal shows the three inputs for each model.

### GWAS for SLB resistance in the NAM panel

**Figure 3:**
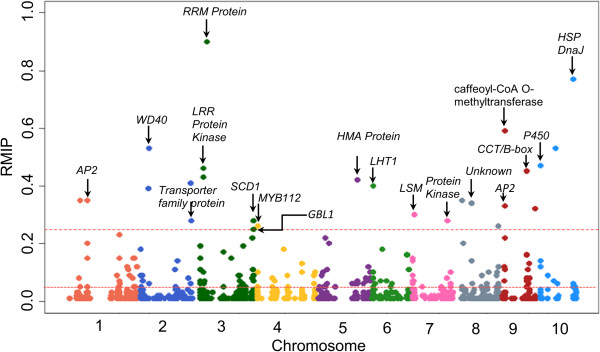
**Manhattan plots from genome-wide association analysis for SLB across the ten chromosome pairs of maize.** The dashed horizontal line in red depicts the resample model inclusion probability (RMIP) thresholds of 0.05 and 0.25. Eighteen candidate genes underlying the most robust GWAS hits (RMIP ≥0.25) located in QTL support intervals are indicated (Table 4).

The updated GWAS (model E, Table 1) was performed using the 28.5 M combined HapMap 1 and 2 SNPs and RDVs with phenotype values adjusted for unlinked QTL from model 7. A total of 192 variants were significantly associated with SLB resistance at RMIP ≥0.05. (Figure 3 and Additional file 1: Table S5). Model 7 QTL support intervals were highly enriched for significant associations: whereas only 17% of all variants tested localized within the QTL support intervals, 98 of 192 (51%) significantly associated variants were in QTL intervals, and 32 out of 33 model 7 QTL support intervals contained one or more of the significant associations. Genes containing or adjacent to the 26 most significantly associated variants (RMIP ≥0.25) were identified (Table 4). Twenty-four candidate genes underlying 25 variants were identified from the B73 reference genome, but no gene was found within 100 kb of SNP S10_64647379 (Table 4). Eighteen of 24 candidate genes were located in model 7 QTL support intervals (Figure 3).

**Table 4:** **Highly significant GWAS variants (RMIP ≥ 0.25) and their adjacent candidate genes**

Chromosome	Physical position (AGPv2)	Allele ^a^	Effect ^b^	***P-***value	RMIP	Genic position	Inside QTL ^c^	Nearest gene ID	Position of nearest gene (AGPv2)		Annotation
									Start	End	
1	59772038	T/C	0.09	9.4E-10	0.35	Intergenic^d^	N	GRMZM2G134671	59764838	59769739	CCT/B-box zinc finger protein
1	90059294	C/T	0.08	8.5E-14	0.35	Intergenic	Y	GRMZM5G806839	90108398	90107598	AP2 domain containing protein
2	34246559	A/C	0.11	1.8E-16	0.39	Intergenic	N	GRMZM5G876621	34361966	34343251	IBR domain-containing protein
2	36938074	G/A	0.10	1.4E-22	0.53	3’UTR	Y	GRMZM2G022627	36936010	36938218	Transducin/WD40 repeat-like superfamily protein
2	204757213	G/A	0.11	4.5E-09	0.41	Intergenic	N	GRMZM2G142932	204771189	204767728	Basic helix-loop-helix (bHLH) DNA-binding protein
2	207520001	CNV-	0.11	4.4E-09	0.28	Intergenic	Y	GRMZM2G062156	207635000	207637178	Transporter family protein
3	16574125	T/C	0.12	1.8E-26	0.43	Non-synonymous_coding^e^	Y	GRMZM2G463580	16575869	16572429	Leucine-rich repeat transmembrane protein kinase
3	16575842	C/G	0.15	9.7E-14	0.46	5’UTR	Y				
3	32885733	C/T	0.19	9.1E-16	0.9	Intergenic	Y	GRMZM2G132936	32957454	32949608	RNA recognition motif (RRM)-containing protein
3	219885527	T/C	-0.11	3.1E-12	0.28	Intronic	Y	GRMZM2G074572	219900229	219884844	Stomatal cytokinesis defective/SCD1 protein
3	220658469	A/C	-0.08	6.0E-12	0.25	Intronic	Y	GRMZM2G130375	220651805	220659525	Beta galactosidase 1
4	2046350	CGG/---	-0.08	3.8E-09	0.26	Non-synonymous_coding	Y	GRMZM2G131442	2044016	2047386	MYB domain protein 112
5	164110001	CNV-	0.10	1.5E-11	0.42	Intergenic	Y	GRMZM2G111872	164120892	164124546	Heavy metal-associated domain containing protein
6	7001531	A/C	-0.08	1.1E-15	0.4	Intergenic	Y	GRMZM2G127342	7036171	7039824	Lysine histidine transporter 1 (LHT1)
7	7958250	+/-	-0.08	1.9E-08	0.3	Intergenic	Y	GRMZM2G021149	7954180	7955128	LSM domain containing protein
7	142997161	G/A	0.09	7.3E-09	0.28	Intergenic	Y	GRMZM2G091919	143003673	143001781	protein kinase superfamily protein
8	22229116	G/A	0.13	1.3E-08	0.35	Intergenic	N	GRMZM2G325612	22249167	22245141	Cytokinin oxidase 5
8	62217745	T/A	0.10	6.3E-14	0.34	Intergenic	Y	GRMZM2G130101	62267220	62273955	Unknown
8	171334544	A/G	-0.06	1.2E-09	0.26	Intronic	N	GRMZM2G476009	171332654	171335508	DNA-directed RNA polymerases subunit RPABC1
9	16263773	A/G	0.17	9.9E-09	0.33	Intergenic	Y	GRMZM2G301860	16253405	16252231	AP2 domain containing protein
9	16317865	G/A	0.10	1.4E-15	0.59	Down_stream	Y	GRMZM2G099363	16320573	16318197	Caffeoyl-CoA O-methyltransferase
9	106051436	G/A	0.08	1.7E-05	0.45	Intergenic	Y	GRMZM2G107886	106201175	106203143	CCT/B-box zinc finger protein
9	139547728	C/G	-0.11	3.4E-08	0.32	Intergenic	N	GRMZM2G402015	139591514	139589475	Plant U-box 13, spotted leaf 11
10	3090835	G/A	-0.13	6.2E-10	0.47	Intergenic	Y	GRMZM2G068465	3066458	3062645	Cytochrome P450
10	64647379	G/A	0.06	5.7E-09	0.53	Intergenic	Y	NA			
10	134501142	C/T	0.06	4.4E-08	0.77	Intronic	Y	GRMZM2G063972	134495450	134504022	Heat shock protein DnaJ

^a^Alleles reported as: “B73 allele/alternate allele”. “CNV-” represents the read depth of a line is significantly lower than B73.

^b^The mean effect of each significant SNP across data subsamples.

^c^SNPs located within model 7 QTL SI are indicated as “Y” and “N” otherwise.

^d^Variants more than 500 bp away from an annotated gene.

^e^Variation lies in the coding region and results in an amino acid change.

### Comparison of multiple NAM GWAS results for SLB resistance

The original NAM GWAS for SLB resistance used a 1106-marker map, model 1 QTL residuals, and 1.6 M HapMap 1 SNPs (GWAS model A, Table 1), and identified 245 significant SNP loci at *p* <1e-04 with RMIP ≥0.05 [8]. Comparing the positions of the 245 SNP associations identified with RMIP ≥0.05 in model A to the 192 variant associations identified in updated model E, only 6% of the total set colocalized within 10-kb windows between the two analyses. The three-fold enrichment of associations within QTL intervals compared to all tested variants observed in the updated GWAS model E (51% of associations vs 17% of all tested) was greater than the two-fold enrichment observed in the original GWAS model A (31% of associations vs 15% of all tested).

To evaluate NAM GWAS sensitivity to different GWAS inputs i.e. genetic map, residual inputs, and GWAS marker density, four separate GWAS models were compared (Table 1). The comparisons of GWAS analyses based on different input data sets indicated that both linkage map density (1106- vs. 7386-marker map) and GWAS marker density (1.6 M SNPs vs. 28.5 M SNPs and RDVs) influenced the GWAS results dramatically (Table 5; Additional file 1: Table S6). When both linkage map and HapMap marker densities were changed simultaneously, less than 25% of associations at RMIP ≥0.05 and 10% of associations at RIMP ≥0.25 localized within 200 kb of each other across analyses (Table 5).

**Table 5:** **Concordance of variants associated with SLB resistance at resample model inclusion probabilities (RMIPs) of 0.05 and 0.25 from five GWAS analyses**

Comparisons	GWAS models ^a^	RMIP ≥ 0.05				RMIP ≥ 0.25			
		No. of all significant SNPs ^b^	Proportion of overlapped SNPs (%) ^c^			No. of all significant SNPs	Proportion of overlapped SNPs (%) ^c^		
			10-kb window	100-kb window	200-kb window		10-kb window	100-kb window	200-kb window
GWAS marker density	B vs. C	326 (151,175)	26	36	38	45 (25,20)	13	13	13
	D vs. E	393 (201,192)	22	34	39	54 (28,26)	11	11	15
Genetic map density	C vs. E	367 (175,192)	14	21	23	46 (20,26)	9	9	9
	B vs. D	352 (151,201)	25	29	35	53 (25,28)	26	28	28
GWAS and genetic marker density	C vs. D	376 (175,201)	13	21	22	48 (20,28)	4	8	8
	B vs. E	343 (151,192)	7	13	15	51 (25,26)	4	4	4
GWAS and genetic marker density and *P*-value	A vs. E	437 (245,192)	6	12	15	52 (26,26)	4	4	4

^a^See Table 1 for details of each GWAS model.

^b^Total number of all the significant SNPs from a pair of analyses is shown outside of parentheses and numbers of significant SNPs from each analysis considered separately are inside the parenthesis.

^c^Proportion of overlapped SNPs was estimated as the total number of overlapped SNPs from a pair of analyses/total number of all the significant SNPs from the two analyses.

Comparisons show the input(s) that differed in each pair of GWAS comparisons.

RMIP values for each variant are determined based on the proportion of data subsamples in which the variant was selected in a multiple regression model at a given *p*-value, so RMIP values are subject to stochastic variation in the random sampling of data sets. Therefore, some of the inconsistency among analyses may be due simply to the process of randomly sampling the complete data set 100 times for each analysis. We estimated the consistency of RMIP values from NAM GWAS (based on re-sampling 80% of RILs) by conducting five separate model E GWAS analyses for all variants on chromosomes 3 and 10, chosen to represent chromosomes with different numbers of QTL. Each of the five analyses included a unique set of 100 random data samples of 80% of RILs to calculate RMIP values from independent runs. Pairwise comparisons of association analyses indicate that 72% - 81% of variants with RMIP ≥0.05, and 88% - 100% of SNPs with RMIP ≥0.25 overlapped within 10-kb windows matched between each sampling procedure (Additional file 1: Table S7). To estimate the consistency of RMIP values with more subsamples, five separate model E GWAS analyses were conducted by analyzing sets of 200 samples and 500 samples on chromosomes 3 and 10. Pairwise comparisons showed that 81% - 89% of associations with RMIP ≥0.05, and 89% - 100% of SNPs with RMIP ≥0.25 overlapped within 10-kb windows when 200 subsamples were used to compute RMIP (Additional file 1: Table S7). About 93% - 96% of variants with RMIP ≥0.05, and 100% of SNPs with RMIP ≥0.25 overlapped within 10-kb windows when 500 subsamples were used (Additional file 1: Table S7).

## Discussion

The maize NAM panel is a community genetic resource for dissecting the genetic architecture of quantitative traits. It allows for the combination of high power in conventional QTL linkage mapping and high resolution in genome-wide association mapping [23, 24]. We identified 33 QTL with small additive effects across 25 NAM families. The 7386-marker map has 6.7 times the marker density of the original 1106-marker map, and, importantly, it has uniform marker spacing at 0.2 cM without gaps. The uniform spacing and denser map improved the power and precision of QTL mapping in our analysis. One of the strongest effect QTL had a discrepancy in position between QTL models 1 and 7 because it localized to a >10 cM gap in the 1106-marker map. The current study resolved what was previously mapped as a single QTL detected at 50.0 cM of chromosome 3 (at the edge of the map gap) into two separate QTL at 43.4 and 54.4 cM. Evidence from fine mapping and high-resolution bi-parental QTL studies [25–27] supports the existence of the two rather than the one QTL. Thus, it appears that the increased map density improved precision of QTL position estimates despite the loss of sample size that occurred by dropping 281 RILs in model 7. This study only evaluated relatively small changes in sample size, we expect much larger effects on results for more substantial sample size changes, as shown by simulation [28].

The high and uniform density of the 7386-marker map eliminates the need for interval mapping, but increases the risk of selecting collinear markers and overfitting the QTL models. In this study, we recognized and corrected collinearity problems that occurred during automated stepwise selection by inspecting results for some diagnostic signatures of collinearity: inflated allele effect estimates at marker pairs within 10 cM of each other, and inflated standard errors of the allele effects. Linkage disequilibrium is extensive within mapping families, such that the increasing power and resolution of QTL mapping plateaus at some point with increasing numbers of markers [29]. We believe that there would be diminishing returns from a more dense linkage map than the current, 0.2 cM dense linkage map (7386-marker map), since we would not expect further QTL resolution and predictive accuracy from more markers, while the collinearity issues and computational burden would continue to increase [30].

Joint linkage–association mapping has been applied in a two-stage process. In the first stage, the phenotype is regressed on genetic markers to identify QTL and estimate their effects. In the second stage, the residual values adjusted by unlinked QTL are then regressed on dense HapMap SNP genotypes, one chromosome at a time. The two-stage approach has several practical advantages in that it is convenient to implement as well as computationally efficient. However, the sensitivity of JLM residual outcomes to first-stage inputs contributes to variation in second-stage GWAS outputs. In this study, changes in inputs (genotypes, phenotypes, sample size, or combinations of them) to the JLM QTL modeling had relatively minor effects on the QTL position estimates (Figure 3). The resulting predicted phenotypic values from the different models had average correlations of *r* =0.95, but the average correlations between corresponding residual values were somewhat lower: *r* =0.82. After removing QTL effects from 9 out of 10 chromosomes, the chromosome-specific residuals are composed of genetic effects (‘signal’) from just one chromosome plus error effects (‘noise’). The chromosome-specific residual values are convenient for SNP testing, because they remove the effects of QTL on other chromosomes, but as a consequence, the residuals represent a lower signal to noise ratio compared to the original phenotypic values. This is unavoidable because each chromosome contributes only a fraction of the total genetic effects to a complex trait. The sensitivity of the residual outcome values to first-stage inputs highlights the difficulty of identifying individual variant effects that account for only a small proportion of the total heritability.

GWAS results were unstable due to changes in the initial inputs to the QTL analysis as well as to the marker set used for association testing. Only about 35% of the associated variants with RMIP ≥0.05 localized to common 100-kb windows between analyses when the HapMap marker set was changed (from 1.6 M SNPs to 28.5 M variants). The proportion of overlapping significant variants in 100-kb windows was even lower (20-30%) when using different genetic maps but the same GWAS markers. Changing both genetic map and GWAS marker inputs reduced the proportion of overlapping significant SNPs to between 13 and 21% (Table 5). Only four candidate genes contained variants that were significant (RMIP ≥0.05) across all four GWAS analyses in a 10-kb window (Additional file 1: Table S5). The generally poor correspondence between GWAS results of the four analyses may be due in part to the highly polygenic nature of the trait. If many sequence variants with small effects control the trait, but only a small proportion of the SNP associations pass stringent thresholds, then relatively small perturbations in analysis inputs could cause substantial differences in the particular SNPs declared as significant.

Eighteen of the 24 candidate genes identified with GWAS model E were in QTL intervals (Table 4). Most of the candidate gene homologs have been reported to be involved in disease resistance (Additional file 1: Table S8). Leucine-rich repeat transmembrane protein kinases (LRR-PK) regulate a wide range of developmental and defense related processes, such as hormone perception, host specific and non-host specific defense response, and wounding response [31]. The well-studied LRR-PK genes include rice *Xa21* (*Xanthomonas resistance 21*) [32, 33], *Arabidopsis FLS2* (*flagellin sensitive 2*) [34], and the *Arabidopsis* elongation factor Tu receptor (EFR) [35]. The *Arabidopsis Cytochrome P450* gene was previously identified as associated with resistance to necrotrophic fungi and aphids [36–38]. The plant U-box 13 (*spotted leaf11*) mutant confers enhanced non race-specific resistance to fungal and bacterial pathogens in rice [39, 40]. The *lysine histidine transporter 1* (*LHT1*) mutant of *Arabidopsis* affects resistance to a broad spectrum of pathogens [41].

The SLB-associated variants identified here by JLM-GWAS were found residing within some QTLs reported in previous studies. Maize genome bins 3.04, 6.01, and 9.02/03 had been identified from different studies contributing to major effects on SLB resistance [25, 42–47]. The association with the highest RMIP (0.9) was localized to 32,885,733 bp of chromosome 3, within a QTL region identified in other populations [42, 47]. The nearest annotated gene to the associated SNP is ~60 kb downstream and encodes an RNA recognition motif (RRM)-containing protein (GRMZM2G132936). Another strong variant association (RMIP = 0.40) was in the *LHT1* gene (GRMZM2G127342) on chromosome 6; this gene was previously suggested to be the causal factor for the classically defined *rhm1* locus based on QTL fine-mapping [46]. One of the most significant SNPs (RMIP = 0.8) is 332 bp downstream of GRMZM2G099363 encoding a caffeoyl-CoA O-methytransferase (CCoAOMT), within a QTL region on chromosome 9 identified in other populations [27, 42]. CCoAOMT has been reported to participate in lignin biosynthesis in plants [48–50]. Lignin has a particular role as a physical barrier against external pathogens, limiting the penetration of pathogens into host cells.

## Conclusion

In conclusion, we recommend use of the updated JLM QTL (model 7) and GWAS (model E) results in the search for candidate genes controlling resistance to Southern leaf blight. The updated QTL model had better prediction accuracy than the original model, and the updated GWAS provided substantially higher marker density, which is expected to provide a better chance of identifying variants in linkage disequilibrium with causal variants. Further work will attempt to validate biologically the effects of candidate genes with the strongest statistical evidence to provide more detailed insight into the genetic basis of SLB resistance. Finally, our results highlight the difficulties and contingencies of reliably identifying genomic variants with small effects on quantitative traits.

## Methods

### Analysis of phenotype data

Kump et al. [8] fit a multivariate (repeated measurements) mixed model to phenotype data collected on resistance of NAM RILs to SLB in three environments. The data and model described by Kump et al. (S1 supplement in [8]) were updated for this study, correcting errors identified in the data file and applying a different approach to modeling the flowering time covariate.

An error was found in the coding levels or dummy values for incomplete blocks nested in population blocks to which entries had been assigned in the 07NC trial. Correcting this led to a significant effect of incomplete blocks in 07NC, which was therefore included in the updated model (BLUPs from the previous analysis are expected to be less precise, because the random effects of incomplete blocks were confounded with the random effect of RILs). There were two additional minor errors that were corrected: i) one plot in 06NC was associated with an incorrect incomplete block; ii) two plots with SLB data from 07NC had their entry information swapped. Instead of fitting the effect of flowering time on disease resistance as a quadratic function for the entire dataset [8], the new model considered the relationship in a more specific manner. In a pre-analysis step of the data for each environment, ANOVA was used to compare the fit of linear, quadratic and cubic functions relating flowering time (measured as days to anthesis) to disease resistance for each rating × environment × population-specific combination. The final multivariate mixed model was as follows:


where **y** = [**y**_1_', **y**_2_']^'^ corresponding to the SLB disease ratings 1 and 2,  corresponding to the overall mean fixed effects for ratings 1 and 2, and so on for the rest of the terms in the model where **ς** = environment fixed effects; **ϕ** = rating x environment x population level flowering time fixed effects (linear or linear + quadratic effects [cubic was never significant in the pre-analysis]); **b** = population block nested in replication × environment random effects; **i** = incomplete block nested in population block × replication × environment random effects; **c** = columns nested in environment random effects; **r** = rows nested in environment random effects; **p** = population random effects; **ς** * **p** = environment × population random effects; **e** = entry (RIL) nested in population random effects; **ς** * **e** = environment × entry (RIL) nested in population random effects; and **ϵ =** residual random effects. As in [8], only those random factors significant (LRT, *p* <0.10) in single environment analyses were retained in the multi-environment model. Modeling of variance-covariance structures was also the same as [8], whereby a bivariate/unstructured covariance was modeled on all random terms in an environment-specific manner for nested design factors (blocking effects, row and column effects, and residual effects) and across all environments for the cross-classified factors (population, population × environment, and entries nested in populations [fitted per population]). For example, the covariance assumed for entries nested in population 1 is as follows, where *i* indicates the specific cohort (*i* = 1 to 28; 1–26 corresponds to the biparental subpopulations of NAM):


The multivariate mixed model was used to estimate BLUPs or phenotypic values as an equally weighted index of the two scores for the first stage analysis of the JLM-GWA procedure. We refer to the BLUPs used in [8] as “original BLUPs” and those calculated for this study as “updated BLUPs.”

### Genotyping and genetic linkage map

NAM RILs were genotyped with the GBS approach by the Institute of Genomic Diversity and the Buckler Lab at Cornell University [15, 16, 51]. A consensus genetic map was constructed based on 7386 SNPs segregating in the NAM RILs. RILs with high levels of homozygosity and reliable genotype scores were used in the construction of the updated genetic map and further genetic analysis. A small proportion of RILs included in the previous analysis [8] were excluded from the current analysis because their GBS data were of insufficient quality to permit reliable genotype calling. A set of markers representing the linkage map at a uniform distance of 0.2 cM was retained from the larger set of GBS SNPs scored, with missing marker data imputed based on linkage intensities and flanking non-missing markers using the Full Sib Family Haplotype Imputation algorithm [52].

### QTL detection and mapping

Joint linkage mapping was performed using stepwise selection implemented in Proc GLMSelect in SAS v9.3. Thresholds for markers to enter and stay in the model at each step were set at α = 0.0001, as used previously [5, 8]. Family main effects were always included and marker effects were nested in families. Kump et al. [8] reported no significant epistatic interactions after accounting for additive effects. We thus modelled the genetic architecture with a pure additive model:


where ***Y*** is an *N* × 1 column vector of the updated SLB resistance best linear unbiased prediction (BLUP) values; ***A*** is an *N* × *P* incidence matrix relating each individual RIL to its corresponding family, ***μ*** is a *P* × 1 column vector of family main effects; ***X***_*i*_ is a *N* × *P* matrix relating each RIL’s genotype score at locus *i* to its corresponding family-specific allele effect, the elements of *X*_*i*_ are coded 0 for lines homozygous for the B73 reference allele, and 2 for homozygotes with the alternate parental allele, 1 for heterozygotes, and a non-integer between 0 and 2 for the imputed recombinants as described above; ***β***_*i*_ is a *P* × 1 column vector of the family-specific additive effects associated with locus *i* relative to B73, *k* is the number of significant loci retained in the final model; and *ϵ* is a *N* × 1 column vector of errors.

High collinearity hinders the selection of markers closest to true QTL positions in linear regression and may result in the selection of pairs of tightly linked loci, with biased effect estimates. Collinearity between tightly linked markers selected in the model was diagnosed based on inflation of standard errors associated with QTL effects and suspiciously large magnitudes of QTL effects of opposite signs for markers located within 10 cM of each other [53]. When obvious collinearity between a pair of markers was detected, one of the problematic predictors was removed from the model, and further selection was implemented with the remaining predictors retained in model. The diagnostic process was repeated until all predictors were free of collinearity.

After initial model selection, the model was further optimized through an iterative process in which one candidate marker was dropped from the full model and replaced with an adjacent marker, and the process was repeated sequentially, fitting each marker within 10 cM of the original peak QTL position one at a time in place of the original marker. The position that resulted in the maximal *R*^*2*^ was recorded. This process was then repeated for each QTL, and then the entire process was iterated until the model stabilized (no marker positions changed). Allele (nested) effects for each QTL within family were estimated in the final optimized QTL model.

To construct support intervals associated with each QTL, a marker immediately adjacent to the QTL on the left side was added to the full model, and the *p*-value for the QTL peak itself was noted. Typically, addition of an adjacent collinear marker reduced the Type III sum of squares for the tested peak QTL marker, resulting in a *p* >0.05 for the peak marker. An iterative process was followed by moving the position of the added marker sequentially along the linkage map to the left side of the QTL peak marker until the tested QTL peak marker became significant at the *p* <0.05. The point at which the QTL peak regained significance signifies the limit of that QTL effect, so the position of the added marker was considered the left boundary of the QTL support interval. The right support interval boundary was identified the same way.

### Internal cross-validation of QTL model prediction accuracy

To compare the prediction accuracies of the models 1and 7 QTL in the NAM panel, we estimated prediction accuracies by a cross validation scheme of random subsampling. The QTL were fixed at the positions selected in the two final models, but we re-estimated the allele effects for the two models in each subsample. Models were compared using a subset of 4354 RILs common between the 4694 RILs used by Kump et al. [8] and the 4413 RILs used in this analysis. Within each replication of the cross-validation, 80% of RILs (3484) of each family were randomly sampled without replacement from the 4354 common RILs to use as a training set. The QTL positions from models 1 or 7 were fit to the data and the QTL allele effects were estimated for each of the two models using their own linkage maps. The remaining 20% of the RILs (870) were held out as a validation set, and the prediction accuracies were estimated as the within-family Pearson’s correlation between predicted values and actual SLB resistance BLUP values. Prediction accuracies were then averaged over 100 random validation sets.

### Correlation analysis of JLM residual outcomes and predicted phenotypic values

GWAS is conducted in the NAM panel on a chromosome-by-chromosome basis; tests for association of SNPs on a particular chromosome are conducted using residuals values from the regression of the SLB phenotypes on the final JLM QTL model after dropping any QTL markers on the chromosome in question (a reduced JLM QTL model). To evaluate the sensitivity of JLM residual outcomes to changes in three JLM inputs, we designed a series of computational experiments controlling for differences in genetic maps, RIL sample sizes and phenotypic data, and combinations of them. A series of six QTL models were constructed with those different inputs. Correlations between the chromosome-specific residual sets were computed for all 15 pairs of model comparisons, as well as for the corresponding predicted phenotypic value sets. Average correlations over 10 chromosomes were computed and compared between residual sets and predicted BLUP values. The QTL peaks and support intervals for those six models involving the sensitivity analysis and model 7 were positioned on the 7386-marker map to examine concordance of the QTL mapping.

### GWAS and identification of candidate genes

The maize HapMap 1 data set includes 1.6 M SNPs polymorphic between B73 and at least one other founder line. We also used an updated version of the maize HapMap 2 data set (dated March 28, 2012), which includes 27.3 M SNPs. About 60% of HapMap 1 SNPs are not included in HapMap2. Therefore, we combined the two SNP data sets and retained 28.2 M unique SNPs. In a few cases, the founder allele calls for the same SNP in the two data sets differed; we retained the HapMap 1 allele calls in such cases, to permit direct comparison to HapMap 1 results. We also included 228,212 read-depth variant (RDV) calls reported by Chia et al. [14], resulting in a total of 28.5 M variants tested for association with SLB resistance. Each read-depth variant represents either an increase or decrease of log2 or more in read depth along a 10 kb window with respect to the reference B73 genome.

The RIL residuals from reduced JLM models (dropping QTL from one particular chromosome) represent the phenotype values for which most of the genetic effects are due to sequence variation on the chromosome considered. GWAS was performed by randomly sampling 80% of the RILs from each family and analyzing chromosome by chromosome with forward stepwise selection of the combined HapMap 1, 2 SNPs and RDVs at *p* <1 × 10^-7^. An exception to this was the original GWAS model A, which used a threshold of *p* <1 × 10^-4^ for inclusion in the chromosome-specific model [8]. The much higher number of variants available for testing in the HapMap 2 data set requires a more stringent threshold to prevent overfitting of GWAS models. This subsampling and analysis procedure was repeated 100 times. The resample model inclusion probability (RMIP) was calculated for each variant as the proportion of 100 data samples in which the variant was selected in the regression model. Candidate genes encompassing or near (within ~100 kb) the variants with strong association signals (RMIP ≥0.25) were identified in the maize B73 genome using the genome browser at http://www.maizegdb.org [54].

### GWAS result comparison

SNPs associated with variation in SLB resistance at two RMIP thresholds (RMIP ≥ 0.05 or RMIP ≥ 0.25) using GWAS model A were compared to those identified with model E. Significant variants from the two GWAS were compared on the basis of their positions on AGP version 2 maize B73 reference genome. Positions of variants identified in different analyses were compared using each of 10-kb, 100-kb and 200-kb windows. The variant match rate was calculated as the ratio of variants from different analyses found within common windows to all significant variants for a pair of analyses. To determine the relative importance of changes in different inputs to GWAS, each combination of genetic map, GWAS marker density, and residual inputs were used to perform separate GWAS analyses, and results were compared on the basis of position matches in windows.

To determine the effect of the number of data resamples on the stability of RMIP estimates, we analyzed 1000 random samples of 80% of all NAM families and performed model E GWAS analyses on chromosomes 3 and 10. We chose chromosomes 3 and 10, as they represent the range of numbers of QTL mapped on each chromosome while greatly reducing the computational burden compared to whole genome analyses. We then compared the consistency of RMIP values from five disjoint samples of 100 resamples each, five disjoint samples of 200 each, and five random partitionings of the 1000 analyses into pairs of 500 resamples. The rate of matching associations at each of 10-kb, 100-kb, and 200-kb windows with RMIP ≥0.05 and RMIP ≥0.25 was computed for all pairwise comparisons of the five association analyses at each sample size.

### Availability of supporting data

The data sets supporting the results of this article are available at the Panzea.org repository, http://panzea.org/db/gateway?file_id=Kump_etal_2011_Nat_Genet_SLB_pheno_data (for raw data) and at the LabArchives.com repository, https://mynotebook.labarchives.com/share/SLB%2520GWAS%2520reanalysis/MjIuMXw0MDg2OC8xNy0yL1RyZWVOb2RlLzc0NDQwODg5fDU2LjE== (for updated BLUPs and SAS codes to perform NAM joint linkage analysis).

## Electronic supplementary material

Additional file 1: **Supplemental Tables S1 – S6.** (XLSX 164 KB)

Additional file 2: Figure S1: Histograms and box plots of prediction correlation coefficients from 100 random cross-validation analyses sets randomly subsampled from the common set of 4354 RILs. (DOCX 52 KB)
