# Supplementary material for: Limits on the reproducibility of marker associations with southern leaf blight resistance in the maize nested association mapping population
Source: BMC Genomics. 2014 Dec 5;15(1):1068. doi: 10.1186/1471-2164-15-1068 (PMC4300987; doi:10.1186/1471-2164-15-1068)
Supplement: Supplementary file 2 — Additional file 2: Figure S1: Histograms and box plots of prediction correlation coefficients from 100 random cross-validation analyses sets randomly subsampled from the common set of 4354 RILs. (DOCX 52 KB) [file 12864_2014_6799_MOESM2_ESM.docx]

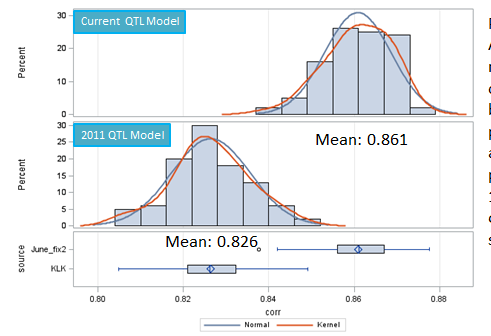


Figure S1. Histograms and box plots of prediction correlation coefficients from 100 random cross-validation analyses sets randomly subsampled from the common set of 4354 RILs. Current QTL Model = Model 7; 2011 QTL Model = Model 1.
